# US Food and Drug Administration Competitive Generic Therapy Approvals and Drug Competition

**DOI:** 10.1001/jamainternmed.2025.6072

**Published:** 2025-11-17

**Authors:** Kevin Kho, Rinku Patel, Harinder Singh Chahal

**Affiliations:** 1 Office of the Commissioner, US Food and Drug Administration, Silver Spring, Maryland; 2 Center for Drug Evaluation and Research, US Food and Drug Administration, Silver Spring, Maryland

## Abstract

This cross-sectional study examines the dynamics of competitive generic therapy drug pricing and volume.

In 2017, Congress established the competitive generic therapy (CGT) pathway to incentivize generic entry when there is inadequate generic competition, ie, there is not more than 1 version (brand or generic) of that drug on the market. Certain CGT-designated products can be eligible for 180 days of marketing exclusivity postapproval, a period during which additional competitor drugs may not be approved but existing competitors are unaffected.^1,2^ Two CGT attributes incentivize quick marketing: first, exclusivity is forfeited if the drug is not marketed within 75 days postapproval (forfeiture does not transfer the exclusivity to another applicant), and second, the US Food and Drug Administration can approve competing applications that duplicate the same branded product until the exclusivity-eligible applicant markets, initiating 180-day of exclusivity.^1^ Prior work demonstrated robust company participation in the CGT pathway; this study analyzes the dynamics of CGT competition, including drug pricing and volume.^1^

## Methods

We first characterized generic drug products approved with CGT exclusivity eligibility from October 1, 2017, through December 31, 2022. This study defines a drug product at the molecule-strength-dosage form level, which is consistent with the product level used for exclusivity purposes; thus, 1 molecule may be marketed in multiple products (eMethods in Supplement 1). Second, for exclusivity-eligible CGTs launching within 75 days with available competitor data pre- and post-CGT entry, we conducted a 25-month event study examining (1) mean prices and volumes of existing drugs 12 months pre-CGT entry and (2) monthly prices and volumes of CGTs, existing drugs, and competitors for 12 months post-CGT entry. As the study did not involve human participants, institutional review board approval was not required.

The price and volume outcomes are ratios of monthly values compared with their respective pre-CGT 12-month averages. A post-CGT entry price level or volume ratio less than 1 indicates a decrease in price or volume, respectively. Transactional volume and sales data were obtained from IQVIA’s National Sales Perspective and adjusted for inflation. Prices exclude manufacturer discounts and rebates.^3^ Analyses were conducted using R, version 4.3.1 (R Foundation).

## Results

Among the 127 CGTs approved with exclusivity eligibility, 106 (83.5%) triggered the exclusivity by marketing within 75 days of approval within a median of 7 days (IQR, 1-41 days) (Table). CGT entry was associated with a reduced price of the median drug by 18% without significantly affecting overall market demand (Table).

**Table.** Characteristics of US Food and Drug Administration–Approved Competitive Generic Therapies (CGTs) With Exclusivity Eligibility From October 2017 to December 2022

Characteristics	No. (%)		Time to market, median (IQR) [range], d	CGTs included in competition analyses, No.^c^	Change in price: ratio of prices after vs before CGT entry, (IQR) [range]^e^	Change in units: ratio of units after vs before CGT entry, (IQR) [range]^f^
	CGT products approved with exclusivity eligibility^a^	CGTs eligible for exclusivity that marketed within 75 d^b^				
Total products	127	106	7 (1-40.8) [0-75]	94	0.82 (0.71-0.94) [0.38-1.9]	1.11 (0.98-1.24) [0.37-5.86]
Complex vs noncomplex^d^						
Complex	21 (16.5)	21 (100)	7 (1-34) [0-69]	20	0.73 (0.67-0.93) [0.38-1.15]	1.33 (1.07-1.67) [0.74-5.84]
Noncomplex	106 (83.5)	85 (80.2)	7 (1-50) [0-75]	74	0.83 (0.73-0.94) [0.58-1.9]	1.09 (0.96-1.18) [0.37-5.86]
Route of administration/dosage						
Intravenous/injectable	34 (26.8)	23 (67.6)	11 (4-55) [0-73]	19	0.93 (0.85-1.03) [0.6-1.9]	1.13 (1.09-1.44) [0.37-5.86]
Ophthalmic/nasal	8 (6.3)	8 (100)	34 (6.8-70.5) [0-75]	6	0.87 (0.72-1.11) [0.54-1.33]	1 (0.79-1.57) [0.72-5.84]
Oral (liquid)	17 (13.4)	14 (82.4)	3.5 (0-20.5) [0-72]	13	0.82 (0.73-0.92) [0.64-0.97]	1.16 (1.07-1.21) [1-1.41]
Oral (solid)	57 (44.9)	50 (87.7)	7 (1-24.2) [0-72]	46	0.76 (0.72-0.9) [0.58-1.12]	1.07 (0.93-1.15) [0.6-3.9]
Topical/transdermal	11 (8.7)	11 (100)	3 (1-38.5) [0-61]	10	0.71 (0.68-0.81) [0.38-0.96]	1.53 (1.26-1.79) [0.96-3.62]
Approval year						
2018	7 (5.5)	7 (100)	11 (3-25.5) [0-34]	7	0.84 (0.68-0.86) [0.64-0.93]	1.09 (0.99-1.1) [0.69-1.12]
2019	22 (17.3)	19 (86.4)	0 (0-5.5) [0-69]	18	0.9 (0.73-0.98) [0.6-1.08]	1.01 (0.85-1.16) [0.63-2.78]
2020	21 (16.5)	16 (76.2)	4.5 (0-31.8) [0-70]	15	0.88 (0.72-0.91) [0.54-1.15]	1.1 (1.04-1.16) [0.74-5.84]
2021	41 (32.3)	37 (90.2)	8 (4-68) [0-75]	29	0.75 (0.72-0.86) [0.61-1.15]	1.1 (1.03-1.22) [0.61-5.86]
2022	36 (28.3)	27 (75.0)	11 (6.5-52.5) [0-73]	25	0.89 (0.73-1.04) [0.38-1.9]	1.29 (1.12-1.65) [0.37-3.9]
Therapeutic area^g^						
Cardiovascular disease	12 (9.4)	10 (83.3)	7 (0-8) [0-28]	10	0.75 (0.73-0.85) [0.58-0.9]	1.13 (1.08-1.17) [0.6-2.55]
Endocrinology, diabetes, and metabolism	15 (11.8)	10 (66.7)	26.5 (5.2-53.8) [0-64]	9	0.76 (0.67-0.92) [0.59-0.97]	1.23 (1.12-1.45) [1.03-1.65]
Infectious disease	16 (12.6)	13 (81.2)	19 (7-69) [0-71]	13	0.8 (0.68-0.93) [0.38-1.1]	1.17 (1.08-1.44) [0.89-3.62]
Medical countermeasure	10 (7.9)	9 (90.0)	4 (2-6) [0-68]	7	0.97 (0.88-1.15) [0.84-1.9]	1.16 (1.13-1.76) [0.37-5.86]
Psychiatry	15 (11.8)	14 (93.3)	1 (0.2-4) [0-61]	13	0.87 (0.68-0.98) [0.61-1.08]	1.05 (0.79-1.09) [0.63-1.31]
All others	59 (46.5)	50 (84.7)	8 (2.2-55) [0-75]	42	0.8 (0.71-0.95) [0.54-1.33]	1.06 (0.93-1.22) [0.61-5.84]

^a^ Column percentage.

^b^ Row percentage.

^c^ Twelve of 106 CGTs with exclusivity eligibility for which the sales data for existing drugs (those on the market before CGT entry) were not available in the drug utilization database, IQVIA, within the 25-month study window, were omitted from the competition analyses conducted using the 25-month event study.

^d^ Difficult to produce small-molecule drugs, such as inhalers, topical products, or extended release injectables, as well as drug-device combinations are considered complex and often have limited competition.

^e,f^ These columns report medians and ranges of product-level measures across all products in the study sample, regardless of CGT designation (ie, these columns include incumbents, CGT drugs, and other generics entering during the study time frame). Each column’s ratio compares a different value (price per extended unit or total volume of product sold in extended units, respectively) after vs before first commercial marketing of the CGT with exclusivity eligibility. Ratios less than 1 indicate that price or volume sold decreased in the months following CGT entry compared with the months before. Conversely, ratios greater than 1 indicate an increase. For example, of the 94 drug products (as defined in the Methods section) in the study sample, the median ratio of post to preperiod prices was about 0.82, meaning that prices for that particular drug product during the postperiod were a mean of 18% lower than during the preperiod.

^g^ Therapeutic areas that had 10 or more products approved with exclusivity eligibility are listed; all remaining products are grouped as “all others.”

Among the 106 marketed CGTs, 94 had analyzable sales data. These drugs entered the market at lower prices than therapeutically equivalent incumbents (Figure, A). By month 12, the weighted mean prices of existing drugs and CGTs were 15.9% and 40.3% lower than average prices pre-CGT entry, respectively.

**Figure.**
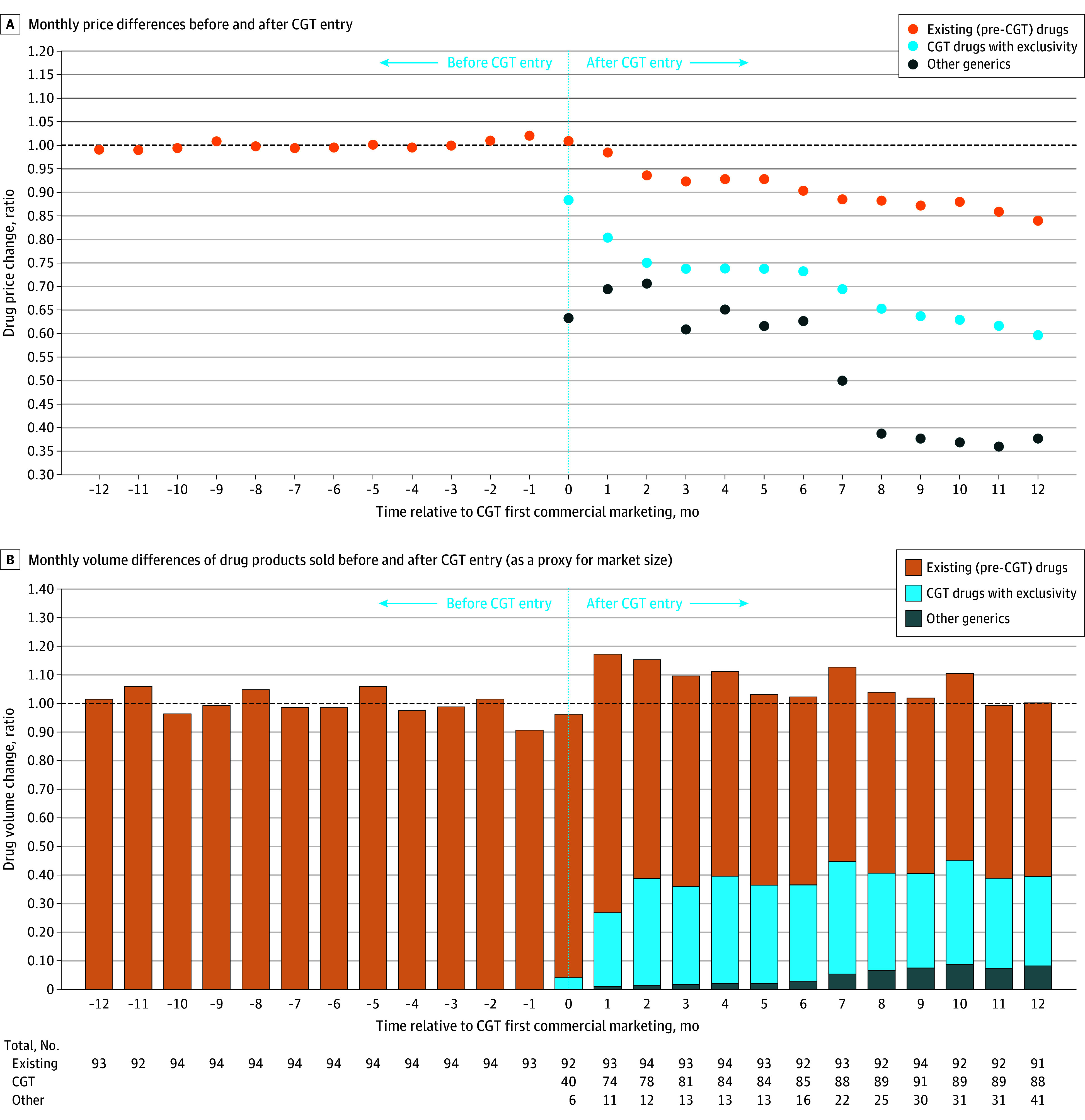
Trends in Monthly Prices and Volumes Among Drug Products 12 Months Before and After Competitive Generic Therapy (CGT) Entry Data were sourced from the IQVIA National Sales Perspectives (NSP), 2017 to 2024. The NSP measures the volume of prescription drug products moving from distributors and manufacturers into various outlets within the retail and nonretail markets and captures approximately 90% of the total pharmaceutical market. Except for the mail channel, these data are estimated based on national projections. Trends in the monthly prices and volumes among drug products in the event study are pictured 12 months before and 12 months after CGT entry relative to their respective pre-CGT 12-month averages. The price level (A) is the post-CGT month’s spending on a drug over spending if bought at pre-CGT average prices. The volume ratio (B) is the post-CGT month’s volume, in units, of a drug, over the average volume pre-CGT entry. For prices and volumes, a ratio of 1 means that there was no difference in either metric before and after CGT entry; while a ratio of less than 1 indicates a decrease in drug price or volume and vice versa for a ratio of greater than 1. In some cases, another manufacturer can enter the market before the CGT product triggers exclusivity, thus the presence of other generics appearing during the CGT exclusivity period. The dip in the price ratio at months 6 and 7 was likely associated with the increased number of products entering the market after the CGT exclusivity expired, as observed in the data table. The number of other generics increased from 13 to 16 in month 6 and 22 in month 7, and this trend was observed through the end of the study period.

Total volume of products sold was relatively consistent over the 25-month period. At the end of the study, existing drugs, CGTs, and other therapeutically equivalent products comprised 61%, 31%, and 8% of market volume, respectively (Figure, B).

## Discussion

A total of 106 of 127 approved CGTs (83%) launched within 75 days, quickly competing with incumbents and lowering prices while capturing market share. In contrast, among certain other non-CGT generics that are first to challenge branded drug patents and are thus eligible for 180-day exclusivity, only 50% launch within 6 months.^4^ The faster CGT entry is likely due to the use or lose nature of CGT exclusivity that only blocks other approvals once triggered by marketing. Further, typically lone generic competitors reduce prices by 31% less than the brand price. The reduction for CGTs is smaller (26.8% by month 6), likely because CGTs are lower-revenue drugs.^5^

This study was limited first by the fact that we could not determine whether the CGT pathway induces entry or is used by manufacturers who would have entered regardless. Second, because our data excluded rebates, the results may understate incumbent responses, as incumbents may have increased rebates alongside price reductions to retain market share.

During the study period, there was no lasting change in total volume of product sold despite increased numbers of manufacturers; existing and CGT products appeared to find an equilibrium to balance competition, market demand, and lower prices. However, a longer-term product-level evaluation is needed to assess whether markets for some CGTs can sustain multiple manufacturers.^6^

## Notes

Supplement 1. eMethods.

Supplement 2. Data sharing statement
