# Supplementary material for: US Food and Drug Administration Competitive Generic Therapy Approvals and Drug Competition
Source: JAMA Intern Med. 2025 Nov 17;186(1):136–8. doi: 10.1001/jamainternmed.2025.6072 (PMC12624475; doi:10.1001/jamainternmed.2025.6072)
Supplement: Supplement 1. — eMethods. [file jamainternmed-e256072-s001.pdf]

Supplemental Online Content

Kho K, Patel R, Chahal HS. US Food and Drug Administration competitive generic therapy approvals and drug competition. *JAMA Intern Med*. Published online November 17, 2025. doi:10.1001/jamainternmed.2025.6072

eMethods.

This supplemental material has been provided by the authors to give readers additional information about their work.

© 2025 Kho K et al. *JAMA Internal Medicine*.

eMethods.

I. Methods for obtaining data for the price and volume analyses

To measure competition in the marketplace, price and volume analyses were conducted on Competitive Generic Therapies (CGTs) approved with exclusivity eligibility from October 2017 through December 2022, as well as their therapeutically equivalent competitor products. The study sample was created using internal FDA records on the generic applications for CGTs, an internal database of cumulative Orange Book listings, and information from the electronic drug registration and listing system (eDRLS).[1, 2]

Product prices (sales dollars divided by extended units) and sales volumes (extended units) were obtained from IQVIA's National Sales Perspective (NSP) dataset, which provides this data by month.[3] Sales dollars in the NSP represent the total amount of sales into the dispensing outlets, chains, and healthcare providers from the manufacturers and distribution centers. Extended units represent the total number of shipped units of a drug – such as, tablets/capsules for solids, grams for creams, milliliters for liquids, etc. When considering a market for therapies with differing dosage forms, IQVIA's extended units variable is an appropriate metric for the purposes of this study to approximate market size in terms of usage (i.e., proxy for patient access).

- The IQVIA National Sales Perspectives™ measures the volume of prescription drug products moving from distributors and manufacturers into various outlets within the retail and non-retail markets. It is the industry standard for measuring pharmaceutical spending because it captures ~90% of the total pharmaceutical market. NSP is used to monitor the actual volume amount of a product that is being distributed in any channel of the pharmaceutical marketplace. Except for the mail channel, these data are estimated based on national projections. Data are available in IQVIA's business intelligence tool SMART for 72-rolling months and are updated monthly.

To ensure appropriate matching between NSP data and the study sample, the CGT product data – at the molecule, strength, dosage form, and applicant level – were matched on national drug codes (NDCs) –

© 2025 Kho K et al. *JAMA Internal Medicine*.

available from NSP and from FDA records associated with each CGT. The prices from the NSP were adjusted for inflation using the Consumer Price Index (CPI) for prescription drugs for all urban consumers (series ID CUSR0000SEMF01), available from the Bureau of Labor Statistics (BLS) website.[4]

The flowchart in the **Supplement Figure** shows how companies' marketing decisions and the data availability led to the final study sample. Beginning with 127 CGTs approved with exclusivity eligibility, our final sample contains 94 of these CGTs along with their competitors.

Note that we treat a molecule approved in several different strengths and dosage forms as multiple drug products. The **Supplemental Table** lists the 65 distinct molecules included in the final sample of 94 drug products. Because other generics duplicating the same branded product can also enter the market in the 12 months after CGT approval, this analysis of the competitive landscape includes any such drugs. For more information on the CGT pathway, including a running list of drugs approved with CGT designation, visit FDA's website: https://www.fda.gov/drugs/generic-drugs/competitive-generic-therapy-approvals.

© 2025 Kho K et al. *JAMA Internal Medicine*.

Supplement Figure: Construction of Competitive Generic Therapy study sample

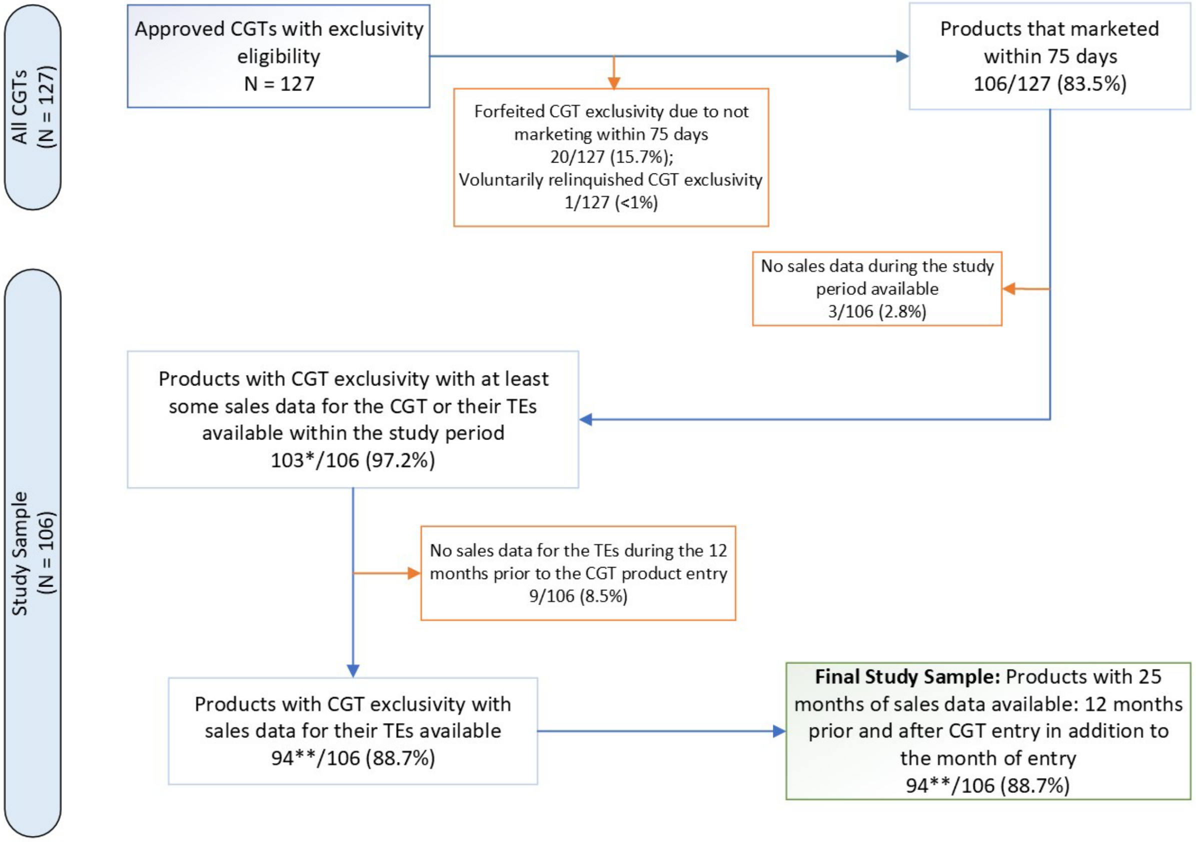

Notes:

CGT: Competitive Generic Therapy. TE: products that are therapeutically equivalent to the products with CGT exclusivity. Sales data refers to data from IQIVIA National Sales Perspective. The study period is specific to each product and corresponds to 12 months prior and after to the month of that CGT product's marketing, as well as the month of marketing for a total of 25 months.

**4 products out of the 103 only had data for the TEs, not the CGT product. This does not exclude them from the sample.*
***3 products out of the 94 only had data for the TEs not the CGT product. This does not exclude them from the sample.*

© 2025 Kho K et al. *JAMA Internal Medicine*.

Supplemental Table: List of the 65 Molecules Included in Final Sample, with Number of Products at the strength and dosage form level for each molecule in parenthesis

Acetaminophen; Butalbital (1)	Hydralazine Hydrochloride; Isosorbide Dinitrate (1)
Aminocaproic Acid (2)	Hydrocortisone Valerate (1)
Amphetamine Sulfate (2)	Iodixanol (2)
Amphotericin B (1)	Isosorbide Dinitrate (1)
Apomorphine Hydrochloride (1)	Levocarnitine (1)
Atropine Sulfate (4)	Levorphanol Tartrate (1)
Bexarotene (1)	Loperamide Hydrochloride (1)
Buprenorphine (1)	Loteprednol Etabonate (1)
Calcitonin Salmon (1)	Mesalamine (1)
Calcium Gluconate (1)	Methylergonovine Maleate (1)
Carboprost Tromethamine (1)	Metyrosine (1)
Cetrorelix Acetate (1)	Mexiletine Hydrochloride (3)
Chlordiazepoxide Hydrochloride; Clidinium Bromide (1)	Morphine Sulfate (2)
Chlorpromazine Hydrochloride (5)	Mupirocin Calcium (1)
Chlorzoxazone (2)	Naloxone Hydrochloride (1)
Clindamycin Phosphate (1)	Nelarabine (1)
Clindamycin Phosphate; Tretinoin (1)	Neostigmine Methylsulfate (1)
Daptomycin (1)	Nitazoxanide (1)
Dexamethasone (3)	Paroxetine Hydrochloride (1)
Diazoxide (1)	Penicillamine (1)
Dicloxacillin Sodium (2)	Phenylephrine Hydrochloride (1)
Dicyclomine Hydrochloride (1)	Potassium Chloride (2)
Digoxin (2)	Pyridostigmine Bromide (1)
Dihydroergotamine Mesylate (1)	Pyrimethamine (1)
Diltiazem Hydrochloride (3)	Sildenafil Citrate (1)
Doxepin Hydrochloride (1)	Sucralfate (1)
Erythromycin (2)	Tazarotene (2)
Erythromycin Ethylsuccinate (1)	Theophylline (1)
Estradiol (1)	Thiothixene (4)
Ethinyl Estradiol; Norelgestromin (1)	Timolol Maleate (3)
Fenoprofen Calcium (1)	Vigabatrin (1)
Foscarnet Sodium (1)	Zinc Sulfate (2)
Fosfomycin Tromethamine (1)	

© 2025 Kho K et al. *JAMA Internal Medicine*.

II. Methods for calculating the outcome measures in the 25-month event study

1. Outcome 1: Changes in drug prices

To measure overall changes in price for the study drugs, we calculated the price level in each month—the ratio of that month's expenditure on drugs in the sample to what the same volume of those drugs would have cost if purchased at their original average prices before CGT entry.

First, for each drug, as a baseline, we calculated the average price during the 12-month period before CGT entry. If a drug was only sold in some of the 12 months leading up to CGT entry, then its baseline price is its average price over those months when it was sold.

In each month, we considered baseline cost of a drug to be what that month's volume would have cost using the baseline price (i.e., current units x baseline price). In a given month, the baseline cost of all N drugs in our sample was the sum:

$$\sum_{i=1}^{N}(units_i * baselinePrice_i)$$

Thus, in each month, the overall price level for all N drugs in the sample is the following ratio of the current, actual expenditure to the baseline cost:

$$\frac{\sum_{i=1}^{N}(units_i * price_i)}{\sum_{i=1}^{N}(units_i * baselinePrice_i)}$$

2. Outcome 2: Changes in drug volume

To measure changes in volume for each drug, we calculated in each month the ratio of that month's volume in extended units to the average monthly volume during the 12 months leading up to CGT entry. Drug volume is a proxy for patient access to CGT products, with an increase in the ratio indicating improved availability and vice versa.

© 2025 Kho K et al. *JAMA Internal Medicine*.

First, for each drug, as a baseline, we calculated the average monthly volume during the 12-month period before CGT entry. If a drug was only sold in some of the 12 months leading up to CGT entry, then its baseline volume is its average volume over those months when it was sold. The monthly volume ratio for each drug is thus:

$$\frac{volume}{baselineVolume}$$

In each month, the overall volume measure for all N drugs in our sample is the average of these individual ratios, weighted by the individual drugs' average monthly expenditures during the 12-month period leading up to CGT entry:

$$\sum_{i=1}^{N} \omega_i \frac{volume_i}{baselineVolume_i}$$

where,

$$\omega_i = \frac{baselineMonthlyExpenditure_i}{\sum_{j=1}^{N} baselineMonthlyExpenditure_j}$$

Thus, the overall volume measure accounts for the economic significance of the drugs in the study sample relative to one another in terms of expenditure prior to CGT entry.

III. References:

1. U.S. Food and Drug Administration. *Electronic Drug Registration and Listing System (eDRLS)*. 2024. Last Accessed: June 18, 2024. Available from: https://www.fda.gov/drugs/guidance-compliance-regulatory-information/electronic-drug-registration-and-listing-system-edrls.
2. U.S. Food and Drug Administration. *Orange Book: Approved Drug Products with Therapeutic Equivalence Evaluations*. 2024. Last Accessed: June 18, 2024. Available from: http://www.accessdata.fda.gov/scripts/cder/ob/default.cfm.
3. IQVIA. *SMART – US Edition, National Sales Perspectives™ online tool.* 2024. Last Accessed: April 30, 2024. Available from: https://www.iqvia.com/.
4. U.S. Bureau of Labor Statistics. *Consumer Price Index for All Urban Consumers (CPI-U): Prescription drugs*. 2024. Last Accessed: April 30, 2024. Available from: https://beta.bls.gov/dataViewer/view/timeseries/CUSR0000SEMF01;jsessionid=A3FD89CB938E995BD3A026E078F4532D.

© 2025 Kho K et al. *JAMA Internal Medicine*.
