# Supplementary material for: US Food and Drug Administration Competitive Generic Therapy Approvals and Drug Competition
Source: JAMA Intern Med. 2025 Nov 17;186(1):136–8. doi: 10.1001/jamainternmed.2025.6072 (PMC12624475; doi:10.1001/jamainternmed.2025.6072)
Supplement: Supplement 2. — Data sharing statement [file jamainternmed-e256072-s002.pdf]

Data Sharing Statement

Data

Data available: No

Additional Information

Explanation for why data not available: Much of the data used is internal to FDA or under contract from third parties. We have linked to publicly available data.
